# MV130 in the Prevention of Recurrent Respiratory Tract Infections: A Retrospective Real-World Study in Children and Adults

**DOI:** 10.3390/vaccines12020172

**Published:** 2024-02-07

**Authors:** Karla Montalbán-Hernández, Ana Cogollo-García, Patricia Girón de Velasco-Sada, Raquel Caballero, Miguel Casanovas, José Luis Subiza, Laura Conejero

**Affiliations:** Inmunotek S.L., 28805 Madrid, Spain; kmontalban@inmunotek.com (K.M.-H.); acogollo@inmunotek.com (A.C.-G.); pgiron@inmunotek.com (P.G.d.V.-S.); rcaballero@inmunotek.com (R.C.); mcasanovas@inmunotek.com (M.C.);

**Keywords:** MV130, trained immunity-based vaccines, respiratory tract infections, recurrent respiratory tract infections, upper respiratory tract infections, lower respiratory tract infections, real world study

## Abstract

Respiratory tract infections (RTIs) are among the most common and important problems in clinical medicine, making antibiotics the gold standard therapeutic option regardless of their frequent viral etiology. Their excessive and inappropriate use contributes to the rapid rise of antibiotic resistance and underscores the need for alternative strategies, especially when dealing with recurrent RTIs. Prevention is the ideal alternative, but specific vaccines targeting a wide range of respiratory pathogens are scarce. MV130 is a sublingual bacterial vaccine that induces trained immunity and provides non-specific protection against respiratory pathogens in various clinical settings according to the concept of TIbV (Trained Immunity-based Vaccine). A retrospective real-world study (RWS) was conducted to evaluate the annual incidence of RTIs and the consumption of antibiotics before and after the administration of MV130, using data sourced from the medical records of 599 patients (186 children and 413 adults) who suffered from recurrent RTIs. The median number of infectious episodes in children was significantly reduced by more than 70% from 5 episodes (interquartile range (IQR) 4.0–6.0) to 1 (IQR, 0.0–2.0) (*p* < 0.001) after MV130. Similarly, in adults, the median number of episodes before MV130 immunization was 5 (IQR, 4.0–6.0), which dropped by more than 80% to 1 (IQR, 0.0–1.0) during the year following MV130 immunization (*p* < 0.001). The median number of antibiotic courses also significantly decreased for both children and adults by over 80% (*p* < 0.001). This RWS showed that MV130 is an effective strategy for the prevention of respiratory infections and the reduction of associated antibiotic consumption.

## 1. Introduction

Respiratory tract infections (RTIs) are a prevalent and diverse group of illnesses affecting the upper and lower respiratory tract, from mild illnesses like the common cold to severe and potentially life-threatening diseases such as influenza or COVID-19. Due to their high prevalence and the variety of pathogens involved, these infections are a major public health concern and remain a leading cause of death worldwide [1]. RTIs can be caused by a wide range of pathogens, including viruses, bacteria, and occasionally fungi, making their etiology multifaceted. The fact that certain infections (e.g., viral) can predispose individuals to others (e.g., bacterial) adds to this complexity. Upper respiratory tract infections (URTIs), which include rhinitis, pharyngitis, tonsillitis, and otitis media, account for 88% of total respiratory infections and cause mild to moderate symptoms [1]. Lower respiratory tract infections (LRTIs), which include acute bronchitis, bronchiolitis, and pneumonia, are less frequent but cause the most severe illnesses [2,3].

RTIs can become recurrent in certain individuals. Recurrent RTIs (RRTIs) account for episodes that are long-lasting, occur repeatedly over time, are associated with unusual complications, or are not solved with current treatments [4]. Children, the elderly, and individuals with compromised immune systems are particularly vulnerable [4]. Viruses such as the respiratory syncytial virus, influenza virus, and rhinovirus, among others, are the main causative agents responsible for RRTIs [5,6,7], although secondary bacterial infections are associated with severe clinical complications [8]. Bacterial infections are observed in 60% of patients with RTI symptoms lasting for 10 days or more [9,10]. Amongst bacteria, the most common are *S. pneumoniae*, *H. influenzae*, *M. catarrhalis*, and *S. pyogenes* [11].

Antibiotics are considered the mainstay treatment for RTIs worldwide, despite the viral etiology of many of these processes. Moreover, in most cases, they are prescribed empirically without knowing the sensitivity of the causative pathogen [12]. This leads to treatment failures and negative collateral consequences, such as adverse reactions and/or the selection of antibiotic-resistant bacteria, a serious global threat [13,14,15,16,17]. In the case of patients experiencing RRTIs, this becomes more pronounced. Therefore, it is essential to have alternatives for managing this type of infection, particularly for individuals who frequently suffer from recurrent infections [18].

Prevention strategies for RTIs are limited due to the high number of pathogens causing them and the restricted availability of pathogen-specific vaccines [19]. In recent years, however, new concepts about the training and memory capacity of the innate immune system have emerged, offering the potential to develop broad-spectrum vaccines [20]. These vaccines, known as TIbVs (Trained Immunity-based Vaccines), may consist of bacterial components but can provide protection against numerous bacteria, fungi, or viruses [20,21,22,23]. Trained immunity is characterized by the long-term functional reprogramming of innate immune cells [24]. This training process leads to an enhanced innate immune response upon secondary stimulation, increasing the ability to eliminate infections caused by unrelated pathogens not included in the TIbV [25,26].

MV130 is a sublingual vaccine composed of whole-cell heat-inactivated bacteria that has been shown to induce trained immunity [22] and is classified as a TIbV [20]. Mucosal vaccines have the potential to induce robust protective mucosal immunity at the site of infection, making them a strong alternative candidate to parenteral vaccines. The latter, despite inducing systemic immunity, do not regularly trigger a mucosal immune response. In addition, mucosal vaccines have the advantage of non-invasive-needle-free administration [19,27]. In this regard, mucosal immunization with MV130 has been shown to boost cellular and humoral responses in the airways [21,22]. MV130 prevents RTIs in specific clinical settings [28,29] and in randomized controlled trials [30]. To comprehensively assess its potential in the prevention of RTIs, a retrospective real-world study (RWS) was conducted. This study aimed to assess the clinical impact of MV130 prophylaxis on reducing the frequency of infectious episodes and the number of antibiotic courses among children and adults with RRTI in a real-world setting.

## 2. Materials and Methods

### 2.1. RWS Design and Data Collection

A retrospective RWS was conducted using data collected from the medical records of 51 sites (hospitals and clinics) in Spain, mainly Otolaryngologists or Ear Nose and Throat specialists (ENTs). This study was carried out on a cohort of 599 patients suffering from RRTIs (413 adults and 186 children, age range 5 months–90 years). Data from patients prescribed MV130 as per clinical routine practice between 1 January 2017, and 31 December 2017, were retrieved by physicians from their medical records. RRTIs were considered to have ≥2 infectious episodes per year, as diagnosed by the physician. Inclusion criteria required the complete availability of data on age, gender, type of infection, the number of episodes of RTIs, and the number of courses of antibiotics in the 12 months before and after the initiation of prophylaxis with MV130. No comorbidities were recorded. Because of the retrospective collection of data, there were no bias as to direct or indirect intervention in the prescription of treatment. The evaluation of clinical data consisted of assessing the number of infectious respiratory episodes and courses of antibiotic consumed in the 12 months prior and 12 months after starting the treatment with MV130. Besides, the analysis of all the data, the outcomes for children and adults were analyzed independently. Furthermore, adult and children’s cohorts were subsequently divided into two groups according to whether the patients were diagnosed with URTI or LRTI.

This study, encoded INM-BAC-2017-02, was classified as an Observational Post-authorisation study (EPA-OD) by the Spanish Health Authorities (Agencia Española de Medicamentos y Productos Sanitarios, AEMPS) and was evaluated and approved by the Ethics Committee of the “Comunidad Autónoma de Madrid, Spain”.

### 2.2. MV130 Prophylaxis

MV130 is a mucosal TIbV composed of 90% of selected Gram-positive strains (V101 *Staphylococcus epidermidis*, V102 *S. aureus*, and V104 *Streptococcus pneumoniae*) and 10% Gram-negative strains (V113 *Klebsiella pneumoniae*, V103 *Haemophilus influenzae*, and V105 *Moraxella catarrhalis*). All strains are heat-inactivated whole-cell bacteria at 300 FTU (Formazin Turbidity Units) in 50% glycerol (Inmunotek S.L., Madrid, Spain). MV130 is administered to the sublingual mucosa using a metered pump spray. Posology was 2 daily 100 µL sprays for 3 or 6 months, according to the physician’s criteria.

### 2.3. Statistical Analyses

Excel spreadsheet (365 MSO v2302) (Microsoft; Richmond, VA, USA) with the XLSTAT AddIn (v2022) (Addinsoft; New York, NY, USA) together with GraphPad Prism 9 (v9.5.1) (Dotmatics; Boston, MA, USA) were used for statistical analyses. The Shapiro–Wilk test was used to check whether the data obtained followed a normal distribution. In all cases, it was found that they did not follow a normal distribution.

Descriptive statistics were expressed as the median with the first and third interquartile ranges (IQR). Wilcoxon tests were used for comparative statistics. An estimate of location shift (Hodges–Lehmann) was calculated for the differences between the number of RTIs before and after the initiation of MV130 with the use of IBM SPSS Statistics (v20). A *p*-value of < 0.05 was used as the level of significance. Significant differences were set at * *p* < 0.05, ** *p* < 0.01, and *** *p* < 0.001.

## 3. Results

### 3.1. Patient Characteristics

**Figure 1:**
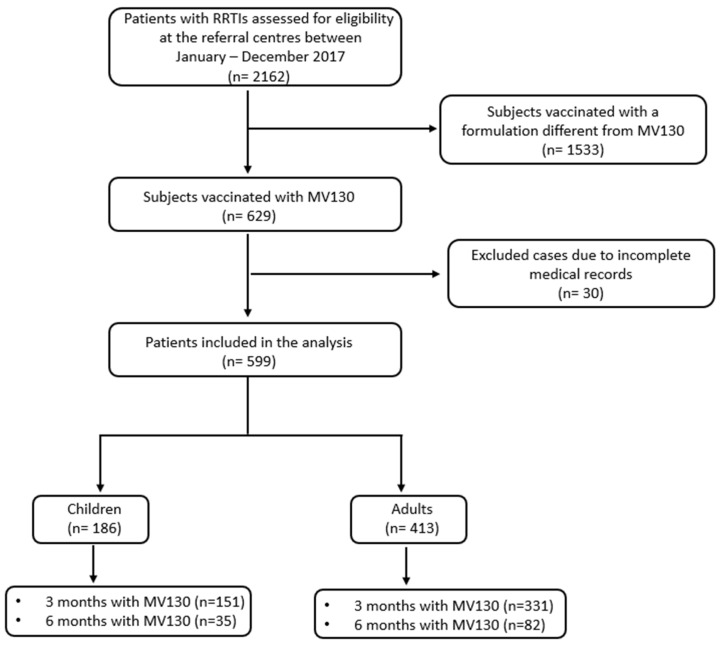
Flow chart of this study. RRTI, recurrent respiratory tract infection.

Data from 599 patients with RRTIs (age range 5 months–90 years) were analyzed. A flow chart of this study is shown (Figure 1). Their demographic and clinical characteristics are shown in Table 1. Out of the total number of patients involved in this study, 413 (69%) were adults, and 186 (31%) were pediatric patients. The number of patients administered MV130 for 3 months was 482 (80%) and 117 (20%) for 6 months. Regarding respiratory pathology, 522 (87%) subjects had recurrent URTIs, and 77 (13%) suffered from recurrent LRTIs. Tonsillitis and bronchitis were the most frequent infections reported in children, whereas tonsillitis and pharyngitis were the most common in adults (Table 1).

### 3.2. MV130 Prophylaxis Reduces the Number of RRTIs

**Figure 2:**
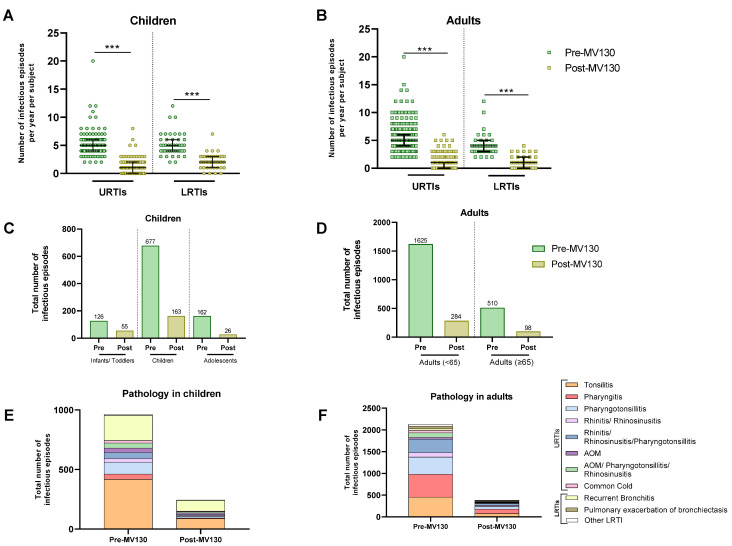
MV130 prophylaxis reduced infectious episodes. The number of URTIs and LRTIs the year before (pre-MV130) and after (post-MV130) initiation with MV130 in children (n = 186) (**A**) and adults (n = 413) (**B**) is shown. Results in the scatter dot plots represent values from single patients; the horizontal lines indicate medians and error bars show the interquartile range. The *p* values were calculated by paired Wilcoxon test. *** *p* < 0.001. Reduction in total number of infectious episodes following MV130 is depicted in bar graphs for all age groups of children (**C**) and adults (**D**). The number of infections in all pathologies from the upper and lower respiratory tracts decreased following MV130 in children (**E**) and adults (**F**). Data are displayed in bars. LRTI, lower respiratory tract infection; URTI, upper respiratory tract infection; AOM, acute otitis media.

The annual number of infectious episodes per subject was recorded both before and after starting MV130 prophylaxis. MV130 was associated with a highly significant reduction in the total number of infectious episodes in both children and adults with URTIs or LRTIs (Figure 2A,B, Table 2 and Table 3) (*p* < 0.001). URTIs were reduced from a median of 5.0 (IQR 4.0–6.0) to 1.0 (IQR 0.0–2.0) (*p* < 0.001) and LRTIs from a median of 5.0 (IQR 4.0–6.0) to 2.0 (IQR 1.0–3.0) (*p* < 0.001) in children (Figure 2A, Table 2). Regarding adults, URTIs were reduced following MV130 from a median of 5.0 (IQR 4.0–6.0) to a median of 1.0 (IQR 0.0–1.0) (*p* < 0.001) and LRTIs from a median of 4.0 (IQR 3.0–5.0) to a median of 1.0 (IQR 0.0–2.0) (*p* < 0.001) (Figure 2B, Table 3). Additionally, adult stratification by age also showed a significant reduction in the number of infectious episodes in all age groups of both upper and lower respiratory tract infections (Supplementary Figure S1A). In children, the total number of infectious episodes dropped from 965 before MV130 administration to 244 after; in adults, it decreased from 2131 to 382 following MV130 administration. The reduction was observed by more than 65% for URTIs and 55% for LRTIs in all three age groups of children (Table 2). This effect was also observed in adults, where the number of infectious episodes also decreased by more than 70% for URTIs and 65% for LRTIs in both younger adults and those aged 65 years and older (Table 3).

Table 2 and Table 3 show the median number of infections [IQR] for each upper or lower respiratory tract, both before and after MV130 prophylaxis. As can be seen, MV130 reduced the total number of infections regardless of pathology, with the most notable reductions in pharyngitis and pharyngotonsillitis in both children and adults.

### 3.3. MV130 Prophylaxis Reduces Antibiotic Consumption

**Figure 3:**
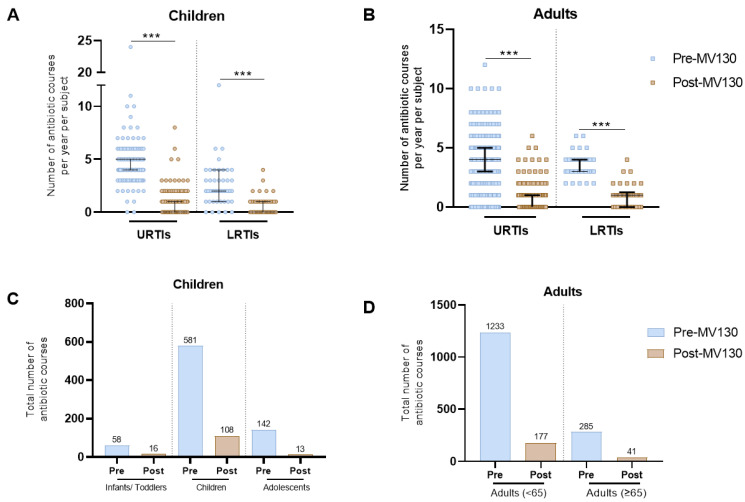
MV130 prophylaxis decreased antibiotic use. The number of antibiotic courses the year before (pre-MV130) and after (post-MV130) initiation with MV130 in children (n = 186) (**A**) and adults (n = 413) (**B**) are shown. Results in the scatter dot plots represent values from single patients; the horizontal lines indicate medians and error bars show the interquartile range. The *P* values were calculated by paired Wilcoxon-test. *** *p* < 0.001. Reduction in total number of antibiotic courses following MV130 is displayed in bar graphs in children (**C**) and adults (**D**). LRTI, lower respiratory tract infection; URTI, upper respiratory tract infection.

Annual antibiotic consumption was assessed based on the total number of antibiotic courses prescribed 12 months before and after starting MV130 prophylaxis. As shown in Figure 3, a highly significant decrease in the total number of prescribed antibiotics was scored in both children and adults following MV130 administration (Figure 3A,B). The accumulative number of antibiotic courses decreased from 781 to 145 in children (Figure 3C) and from 1518 to 218 in adults (Figure 3D). In children, the median number of antibiotic courses per patient prescribed in URTIs significantly decreased from 5.0 (IQR 4.0–5.0) to 0.0 (IQR 0.0–1.0) (*p* < 0.001) and from 2.0 (IQR 1.0–3.5) to 0.0 (IQR 0.0–1.0) (*p* < 0.001) in LRTIs (Figure 3A, Table 4). Similarly, in adults, the median number of antibiotic courses prescribed per patient in URTIs was significantly reduced from 4.0 (IQR 3.0–5.0) to 0.0 (IQR 0.0–1.0) (*p* < 0.001) and in LRTIs from 3.0 (IQR 3.0–4.0) to 1.0 (IQR 0.0–1.0) (*p* < 0.001) (Figure 3B, Table 5). Moreover, adult stratification by age also showed a significant reduction in the number of antibiotic courses prescribed in all age groups for both upper and lower respiratory tract infections (Supplementary Figure S1B). As shown in Supplementary Figure S2, the main antibiotic groups prescribed were beta-lactams for both children and adults. The results show a remarkable decrease in the total number of beta-lactams in both children and adults following MV130 administration. Likewise, the number of macrolides in adults was notably reduced.

## 4. Discussion

There is a recognized public health need for novel vaccine preparations; however, the development of these has been hindered [31]. For this, a full value of vaccines assessment (FVVA) framework has been developed to promote investment in vaccines, which are priorities [31]. In this sense, MV130 is a sublingual polybacterial TIbV that has proven to be effective in reducing recurrent respiratory infections in particularly vulnerable subjects. For instance, it has been beneficial for patients with primary [32] or secondary [33] immunodeficiencies and especially helpful for children prone to bronchiolitis [30]. Since these infections are caused by a wide variety of pathogens, their prevention by specific vaccines is very limited [19]. In this study, we assessed the ability of MV130 to prevent the most common recurrent respiratory infections across a wide demographic spectrum of patients and evaluated its impact on antibiotic consumption in a real-world setting. Even though there is no agreed definition that defines when to consider the number of respiratory infections as recurrent [34], clinicians set two or more episodes from the previous year to be included in this RWS.

The results indicate that MV130 prophylaxis effectively reduced the frequency of most respiratory infections in all age groups. Both children and adults, particularly those suffering from URTIs such as pharyngitis and pharyngotonsillitis, observed about an 80% reduction in infection rates. It should be noted that most patients included in this RWS came from ENT specialists, which could lead to an overrepresentation of URTIs in this study population (87%). However, this percentage is similar to the reported frequency of URTIs with respect to all respiratory infections in the general population [3]. For LRTIs, the data also indicate a notable reduction with MV130 prophylaxis in both children (58%) and adults (71%). In this study cohort, these pathologies were primarily restricted to bronchitis in children and bronchitis and bronchiectasis in adults. Nonetheless, the preventive effect of MV130 against other lower respiratory tract conditions, such as bronchiolitis in young children or pneumonia in adults, had already been noted in previous studies [28,30].

The results in this RWS also show a remarkable decrease in antibiotic consumption (above 85% reduction) following prophylaxis with MV130. These results are consistent with previously published data in vulnerable populations, where a significant reduction in antibiotic consumption was observed with MV130 [32,33,35]. Furthermore, these highlight the common misuse of antibiotic therapy in treating respiratory viral infections [36], a trend that was also evident in the cohort examined in this study. Indeed, most symptomatic pharyngitis and pharyngotonsillitis are considered viral etiologies in all age groups [37,38]. Since there is a strong recommendation to reduce antibiotic use to prevent the selection of resistant bacteria and avoid potential adverse reactions [39], treatments that prevent respiratory infections through antibiotic-free approaches offer additional benefits.

The mechanisms through which MV130 prevents respiratory infections have been explored in experimental viral infection models [21,22], where mucosal immunization with MV130 has proven a strong immune response locally in the airways. By inducing trained immunity, MV130 provides broad protection against different pathogens, including viruses [21,22]. This protection is dependent on the mTOR pathway; it has been demonstrated that MV130 promotes reprogramming of both mouse bone marrow progenitors and in vitro human monocytes. Thus, epigenetic rewiring and metabolic changes are involved in long-term protection against viral infection [22]. In this sense, MV130 can therefore be defined as a vaccine preparation that contains trained immunity inducers in addition to its own antigens [20]. In this regard, BCG, another bacterial vaccine also considered a TIbV [20], has proven effective in preventing respiratory infections of likely viral etiology in the elderly [40] and in reducing viral load after an experimental virus infection in humans [41]. Similarly, a protein-free vaccine composed of three adjuvants has been shown to confer heterologous protection against a number of nosocomial pathogens through the induction of trained immunity [26].

Importantly, innate memory associated with trained immunity may account for a sustained protective effect over several months [42], as demonstrated after MV130 discontinuation in both experimental [22] and clinical settings [28,29]. A long-lasting effect is consistent with the results presented here, as MV130 prophylaxis significantly reduced the number of RTIs beyond the time of administration (3 months in most patients). Intriguingly, the bacteria contained in MV130 possess numerous cross-reactive epitopes with common respiratory viruses [43]. Consequently, a potential antiviral response could complement the non-specific effect of trained immunity, consistent with the notion of TIbVs [20]. In this regard, bacterial respiratory infections may also benefit from the bacterial antigens contained in the MV130 formulation. In this context, MV130 has been described as reducing the need for tonsillectomy, often indicated for recurrent tonsillitis [29], or preventing recurrent pneumonia [28].

As a retrospective RWS, it is limited by the potential for confounding factors and the lack of a control group, although each patient is under their own control. Nonetheless, its strength lies in encompassing nearly 600 patients across all age groups, affected by the most prevalent recurrent respiratory tract infections, and in retrieving homogeneous data in a real-world setting without the constraints imposed by randomized clinical trials. The findings of this RWS, combined with previous research conducted in various clinical contexts, emphasize the clinical advantage of MV130 in preventing these infections while diminishing the related antibiotic usage.

## Figures and Tables

**Table 1:** Demographic and clinical characteristics of this study population ^1^.

Characteristics	All (n = 599)	Adults (n = 413)	Children (n = 186)
	n (%)	n (%)	n (%)
**Gender**			
Male	236 (39)	138 (33)	98 (53)
Female	363 (61)	275 (67)	88 (47)
**Groups of age**			
Infants and toddlers (<2)	26 (4)	NA	26 (14)
Children (2–11)	128 (21)	NA	128 (69)
Adolescents (12–17)	32 (5)	NA	32 (17)
Adults (18–35)	145 (25)	145 (35)	NA
Adults (36–64)	174 (29)	174 (42)	NA
Adults (≥65)	94 (16)	94 (23)	NA
**Length of MV130 immunotherapy**			
3 months	482 (80)	331 (80)	151 (81)
6 months	117 (20)	82 (20)	35 (19)
**Upper Respiratory Infections**	522 (87)	379 (92)	143 (77)
Tonsillitis	183 (31)	101 (25)	82 (44)
Pharyngitis	107 (18)	101 (25)	6 (3)
Pharyngotonsillitis	86 (14)	66 (16)	20 (11)
Rhinitis/rhinosinusitis	27 (5)	21 (5)	6 (3)
Rhinitis/rhinosinusitis/pharyngotonsillitis	64 (11)	54 (13)	10 (5)
AOM	16 (3)	8 (2)	8 (4)
AOM/pharyngotonsillitis/rhinosinusitis	27 (5)	19 (5)	8 (4)
Common cold	12 (2)	9 (2)	3 (2)
**Lower Respiratory Infections**	77 (13)	34 (8)	43 (23)
Pulmonary exacerbation of bronchiectasis	14 (2)	14 (3)	0 (0)
Recurrent bronchitis	51 (9)	10 (2)	41 (22)
Other lower respiratory infections	12 (2)	12 (3)	2 (1)

^1^ Data are presented as numbers (%). The pediatric population, which comprises infants and toddlers, children, and adolescents, will be referred to as children throughout the text; AOM: Acute Otitis Media.

**Table 2:** Median number of infectious episodes before and after MV130 prophylaxis in children.

	Number of Subjects	Episodes (n)		Median [IQR] per Subject				
		Pre	Post	Pre	Post	P (Wilcoxon)	H-L	% Decrease in the Number of Episodes
**Upper Respiratory Infections**	**143**	**744**	**151**	**5.0 (4.0, 6.0)**	**1.0 (0.0, 2.0)**	**<0.001**	**−** **4.0 (** **−** **4.5,** **−** **4.0)**	**80%**
Tonsillitis	82	419	87	5.0 (4.0, 6.0)	1.0 (0.0, 2.0)	<0.001	−4.0 (−4.5, −4.0)	79%
Pharyngitis	6	43	6	5.0 (4.3, 5.0)	1.0 (0.3, 1.0)	0.027	−4.0 (−12.5, −2.0)	86%
Pharyngotonsillitis	20	99	15	4.0 (4.0, 6.0)	1.0 (0.0, 1.0)	<0.001	−4.0 (−0.5, −3.0)	85%
Rhinitis/Rhinosinusitis	6	31	9	5.0 (3.3, 6.0)	1.5 (1.0, 2.0)	0.027	−3.0 (−6.5, −1.5)	71%
Rhinitis/rhinosinusitis/Pharyngotonsillitis	10	52	7	5.0 (4.3, 5.8)	1.0 (0.0, 1.0)	0.005	−4.5 (−5.0, −4.0)	87%
AOM	8	36	12	4.5 (4.0, 5.0)	2.0 (1.0, 2.0)	0.011	−3.0 (−4.0, −2.0)	67%
AOM/Pharyngotonsillitis/Rhinosinusitis	8	43	10	5.0 (4.8, 5.0)	0.5 (0.0, 2.0)	0.017	−4.0 (−6.0, −2.0)	77%
Common cold	3	21	5	8.0 (6.5, 8.0)	2.0 (1.5, 2.0)	0.103	−5.5 (−6.0, −4.0)	76%
**Lower Respiratory Infections**	**43**	**221**	**93**	**5.0 (4.0, 6.0)**	**2.0 (1.0, 3.0)**	**<0.001**	**−** **3.0 (** **−** **3.5,** **−** **2.5)**	**58%**
Recurrent bronchitis	41	211	90	5.0 (4.0, 6.0)	2.0 (1.0, 3.0)	<0.001	−2.5 (−3.5, −2.0)	57%
Other infections of the lower respiratory tract	2	10	3	5.0 (4.5, 5.5)	1.5 (1.3, 1.8)	0.178	−3.5 (−4.0, −3.0)	70%
**All**	**186**	**965**	**244**	**5.0 (4.0, 6.0)**	**1.0 (0.0, 2.0)**	**<0.001**	**−** **4.0 (** **−** **4.5,** **−** **2.5)**	**75%**

AOM: Acute Otitis Media.

**Table 3:** Median number of infectious episodes before and after MV130 prophylaxis in adults.

	Number of Subjects	Episodes (n)		Median [IQR] per Subject				
		Pre	Post	Pre	Post	P (Wilcoxon)	H-L	% Decrease in the Number of Episodes
**Upper Respiratory Infections**	**379**	**1986**	**340**	**5.0 (4.0, 6.0)**	**1.0 (0.0, 1.0)**	**<0.001**	**−** **4.0 (** **−** **4.5,** **−** **4.0)**	**83%**
Tonsillitis	101	455	79	4.0 (3.0, 5.0)	0.0 (0.0, 1.0)	<0.001	−3.5 (−4.0, −3.5)	83%
Pharyngitis	101	528	102	5.0 (4.0, 6.0)	1.0 (0.0, 2.0)	<0.001	−4.0 (−4.5, −3.5)	81%
Pharyngotonsillitis	66	392	59	6.0 (4.0, 7.0)	1.0 (0.0, 1.0)	<0.001	−5.0 (−5.5, −4.5)	85%
Rhinitis/Rhinosinusitis	21	103	26	4.0 (3.0, 6.0)	1.0 (1.0, 2.0)	<0.001	−3.5 (−4.5, −2.5)	75%
Rhinitis/rhinosinusitis/Pharyngotonsillitis	54	310	45	5.0 (4.0, 7.0)	1.0 (0.0, 1.0)	<0.001	−4.5 (−5.0, −4.0)	85%
AOM	8	42	11	5.0 (3.8, 5.8)	1.5 (1.0, 2.0)	0.011	−3.7 (−6.0, −2.0)	74%
AOM/Pharyngotonsillitis/Rhinosinusitis	19	104	11	5.0 (4.0, 7.0)	0.0 (0.0, 1.0)	<0.001	−5.0 (−5.5, −4.0)	89%
Common cold	9	52	7	6.0 (5.0, 6.0)	1.0 (0.0, 1.0)	0.007	−5.0 (−6.0, −4.0)	87%
**Lower Respiratory Infections**	**34**	**145**	**42**	**4.0 (3.0, 5.0)**	**1.0 (0.0, 2.0)**	**<0.001**	**−** **3.0 (** **−** **3.5,** **−** **2.5)**	**71%**
Pulmonary exacerbations of bronchiectasis	14	62	22	4.0 (3.0, 4.8)	1.0 (1.0, 2.8)	0.001	−2.5 (−3.5, −1.5)	65%
Recurrent bronchitis	10	41	14	4.0 (3.3, 4.0)	1.0 (1.0, 2.0)	0.005	−2.5 (−3.5, −2.0)	66%
Other infections of the lower respiratory tract	10	42	6	3.5 (2.3, 5.0)	0.0 (0.0, 1.0)	0.005	−3.5 (−5.0, −2.5)	86%
**All**	**413**	**2131**	**382**	**5.0 (4.0, 6.0)**	**1.0 (0.0, 1.0)**	**<0.0001**	**−** **4.0 (** **−** **4.5,** **−** **4.0)**	**82%**

AOM: Acute Otitis Media.

**Table 4:** Median number of antibiotic courses before and after MV130 prophylaxis in children.

	Number of Subjects	Antibiotic Courses (n)		Median [IQR] per Subject				
		Pre	Post	Pre	Post	P (Wilcoxon)	H-L	% Decrease in the Number of Antibiotic Courses
**Upper Respiratory Infections**	**143**	**671**	**115**	**5.0 (4.0, 5.0)**	**0.0 (0.0, 1.0)**	**<0.001**	**−3.0 (** **−3.5,** **−3.0)**	**83%**
Tonsillitis	82	393	72	5.0 (4.0, 5.0)	0.0 (0.0, 1.0)	<0.001	−4.0 (−4.5, −3.5)	82%
Pharyngitis	6	41	6	4.0 (3.3, 4.8)	1.0 (0.3, 1.0)	0.043	−3.0 (−14, −0.5)	85%
Pharyngotonsillitis	20	76	6	4.0 (3.0, 4.0)	0.0 (0.0, 1.0)	<0.001	−3.5 (−4.0, −2.5)	92%
Rhinitis/Rhinosinusitis	6	24	5	3.0 (3.0, 3.8)	1.0 (0.3, 1.0)	0.028	−2.5 (−5.5, −1.5)	79%
Rhinitis/rhinosinusitis/Pharyngotonsillitis	10	46	4	5.0 (4.0, 5.0)	0.0 (0.0, 1.0)	0.005	−4.5 (−5.0, −3.5)	91%
AOM	8	36	12	4.5 (4.0, 5.0)	2.0 (1.0, 2.0)	0.012	−3.0 (−4.0, −2.0)	67%
AOM/Pharyngotonsillitis/Rhinosinusitis	8	46	10	5.5 (4.8, 6.3)	0.5 (0.0, 2.0)	0.018	−4.5 (−6.5, −2.5)	78%
Common cold	3	9	0	3.0 (3.0, 3.0)	0.0 (0.0, 0.0)	0.109	−3.0 (−3.0, −3.0)	100%
**Lower Respiratory Infections**	**43**	**110**	**30**	**2.0 (1.0, 3.5)**	**0.0 (0.0, 1.0)**	**<0.001**	**−1.5 (** **−2.0,** **−1.5)**	**73%**
Recurrent bronchitis	41	103	27	2.0 (1.0, 3.0)	0.0 (0.0, 1.0)	<0.001	−1.5 (−2.0, −1.5)	74%
Other infections of the lower respiratory tract	2	7	3	3.5 (3.3, 3.8)	1.5 (1.3, 1.8)	0.180	−2.0 (−2.0, −2.0)	57%
**All**	**186**	**781**	**145**	**4.0 (3.0, 5.0)**	**0.0 (0.0, 1.0)**	**<0.001**	**−3.5 (** **−3.5,** **−3.0)**	**81%**

AOM: Acute Otitis Media.

**Table 5:** Median number of antibiotic courses before and after MV130 prophylaxis in adults.

	Number of Subjects	Antibiotic Courses (n)		Median [IQR] per Subject				
		Pre	Post	Pre	Post	P (Wilcoxon)	H-L	% Decrease in the Number of Antibiotic Courses
**Upper Respiratory Infections**	**379**	**1400**	**185**	**4.0 (3.0, 5.0)**	**0.0 (0.0, 1.0)**	**<0.001**	**−** **4.0 (** **−** **4.0,** **−** **3.5)**	**87%**
Tonsillitis	101	425	64	4.0 (3.0, 5.0)	0.0 (0.0, 1.0)	<0.001	−3.5 (−4.0, 3.5)	85%
Pharyngitis	101	272	37	3.0 (2.0, 3.0)	0.0 (0.0, 0.0)	<0.001	−2.5 (−2.5, −2.0)	86%
Pharyngotonsillitis	66	269	33	4.0 (3.0, 5.0)	0.0 (0.0, 1.0)	<0.001	−3.5 (−4.0, −3.0)	88%
Rhinitis/Rhinosinusitis	21	67	16	3.0 (2.0, 4.0)	1.0 (0.0, 1.0)	<0.001	−2.0 (−3.0, −1.0)	76%
Rhinitis/rhinosinusitis/Pharyngotonsillitis	54	247	26	4.0 (4.0, 5.0)	0.0 (0.0, 1.0)	<0.001	−4.0 (−4.0, −3.5)	89%
AOM	8	37	4	3.5 (2.8, 5.8)	0.0 (0.0, 1.0)	0.012	−3.8 (−6.5, −2.0)	89%
AOM/Pharyngotonsillitis/Rhinosinusitis	19	58	2	4.0 (2.0, 4.0)	0.0 (0.0, 0.0)	<0.001	−3.0 (−4.0, −2.0)	97%
Common cold	9	25	3	3.0 (1.0, 3.0)	0.0 (0.0, 1.0)	0.008	−2.0 (−3.5, −1.5)	88%
**Lower Respiratory Infections**	**34**	**118**	**33**	**3.0 (3.0, 4.0)**	**1.0 (0.0, 1.0)**	**<0.001**	**−** **2.5 (** **−** **3.0,** **−** **2.0)**	**72%**
Pulmonary exacerbations of bronchiectasis	14	52	20	3.5 (3.0, 4.0)	1.0 (1.0, 2.0)	0.002	−2.5 (−3.0, −1.5)	62%
Recurrent bronchitis	10	35	11	3.0 (3.0, 4.0)	1.0 (1.0, 1.8)	0.005	−2.0 (−3.0, −1.5)	69%
Other infections of the lower respiratory tract	10	31	2	3.0 (2.3, 3.8)	0.0 (0.0, 0.0)	0.005	−3.0 (−3.5, −2.0)	94%
**All**	**413**	**1518**	**218**	**4.0 (3.0, 5.0)**	**0.0 (0.0, 1.0)**	**<0.001**	**−** **3.0 (** **−** **3.0,** **−** **3.0)**	**86%**

AOM: Acute Otitis Media.

## Data Availability

The data presented in this study are available upon request and permission.

## Supplementary Materials

The following supporting information can be downloaded: https://www.mdpi.com/article/10.3390/vaccines12020172/s1, Figure S1: MV130 prophylaxis reduced infectious episodes and antibiotic course prescriptions in adults stratified by age. The number of URTIs and LRTIs (**A**) and antibiotic courses prescribed (**B**) the year before (pre-MV130) and after (post-MV130) initiation with MV130 in adults stratified by age (n = 413) Figure S2: MV130 prophylaxis reduces the main antibiotics used. The number of antibiotic courses divided into groups the year before (pre-MV130) and after (post-MV130) initiation with MV130 in children (n = 186) (**A**) and adults (n = 413) (**B**) is shown.
