# Supplementary figures and images for: MV130 in the Prevention of Recurrent Respiratory Tract Infections: A Retrospective Real-World Study in Children and Adults
Source: Vaccines (Basel). 2024 Feb 7;12(2):172. doi: 10.3390/vaccines12020172 (PMC10893268; doi:10.3390/vaccines12020172)

# Supplementary Figure S1

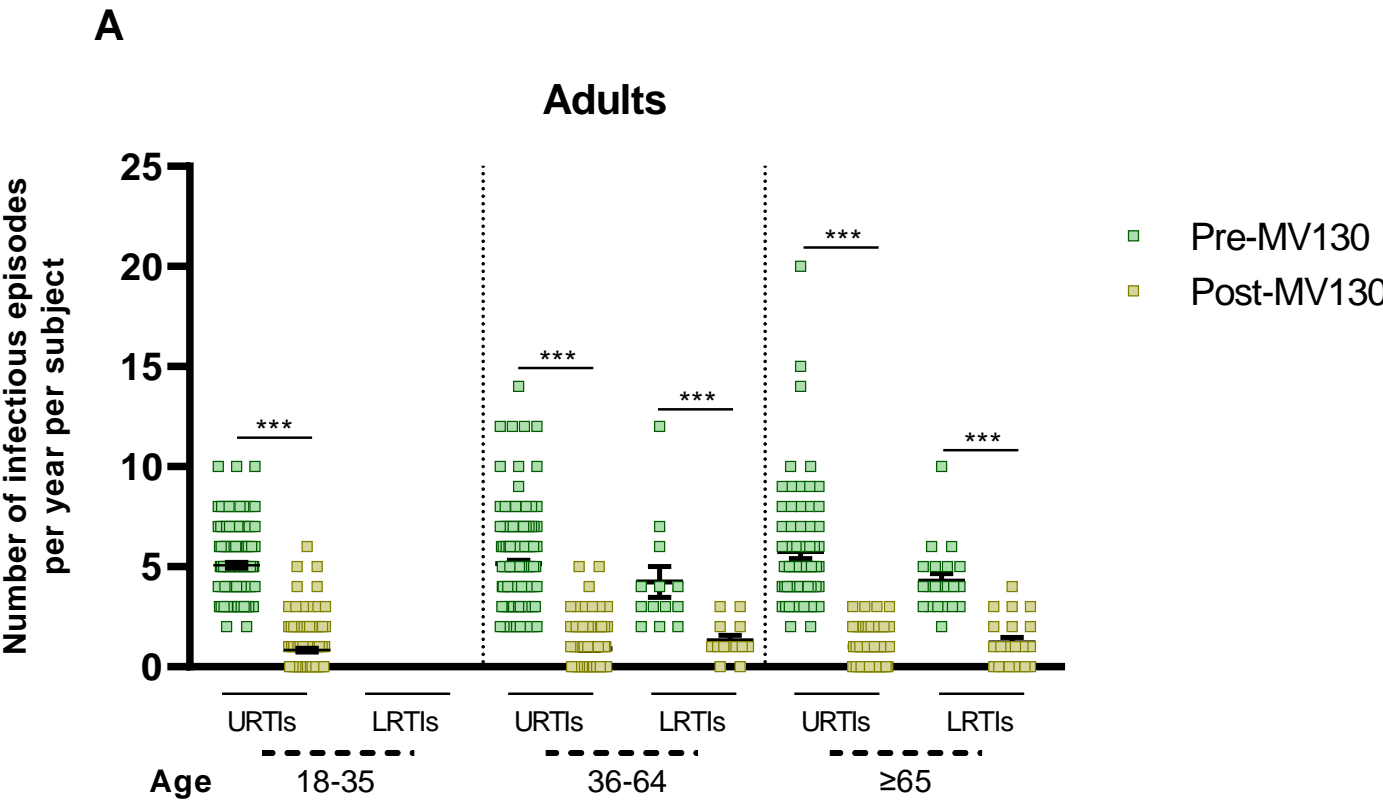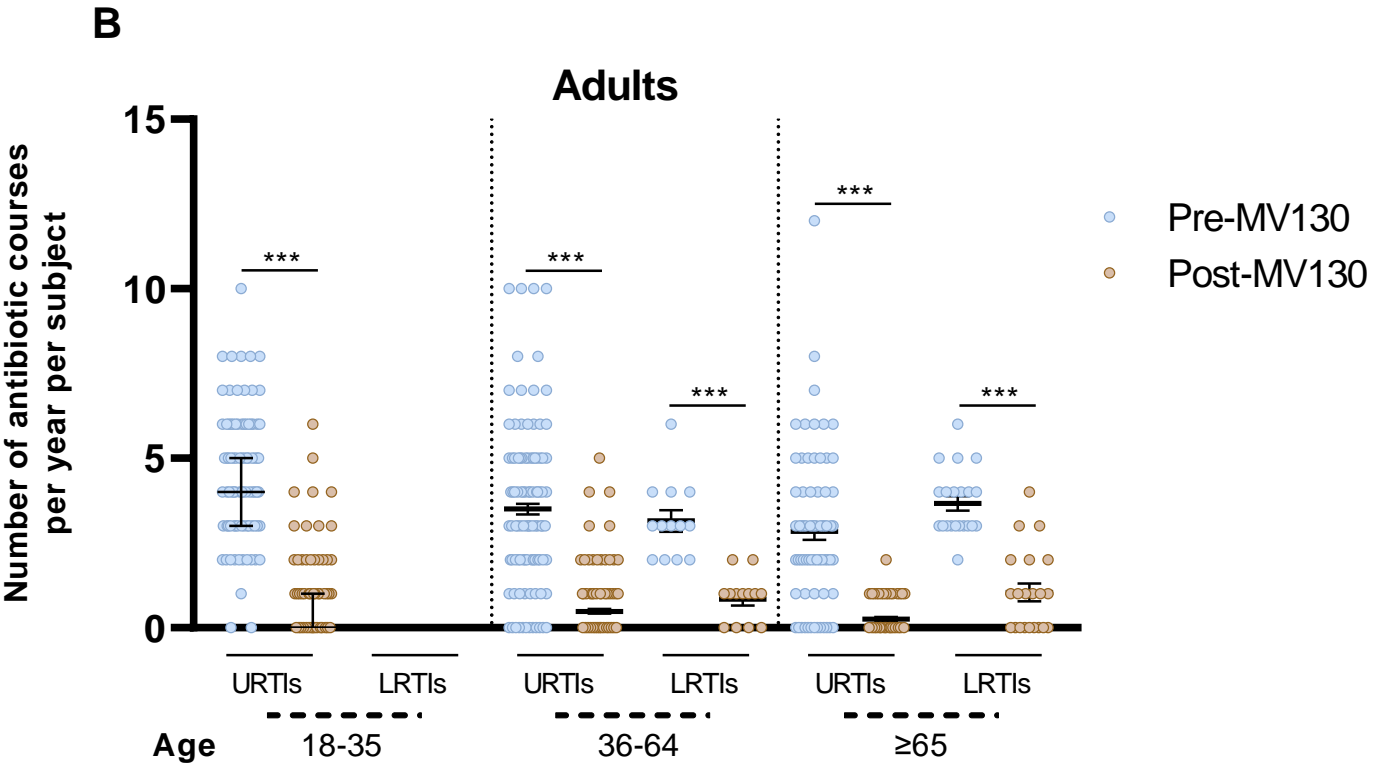

# Supplementary Figure S2

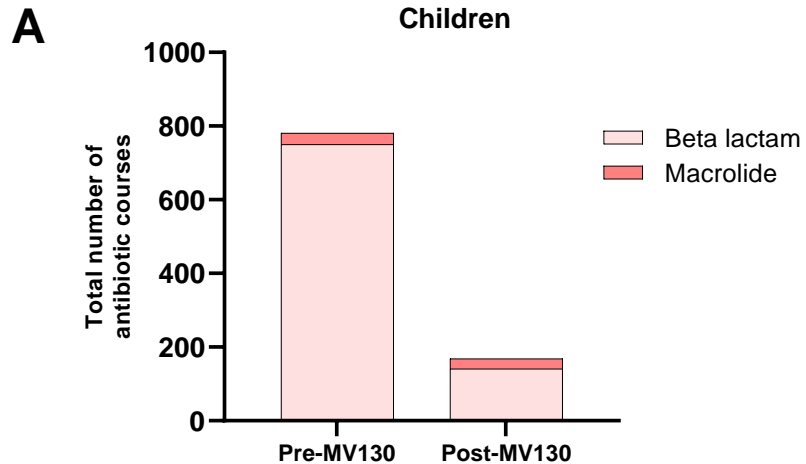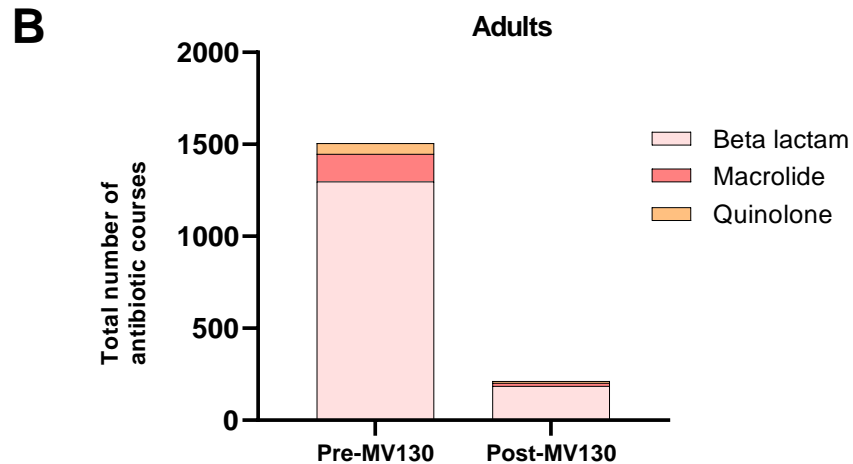

Supplement: Supplementary file 1 [file vaccines-12-00172-s001.zip › vaccines-2785852-supplementary.pdf]
